# MC4R Is Involved in Neuropathic Pain by Regulating JNK Signaling Pathway After Chronic Constriction Injury

**DOI:** 10.3389/fnins.2019.00919

**Published:** 2019-09-10

**Authors:** Yang Zhao, Yan Xin, Haichen Chu

**Affiliations:** ^1^Department of Anesthesiology, The Affiliated Hospital of Qingdao University, Qingdao, China; ^2^Department of Anesthesiology, Qingdao Municipal Hospital, Qingdao, China

**Keywords:** neuropathic pain, melanocortin 4 receptor (MC4R), JNK signaling pathway, chronic constriction injury, nociceptive behavior, paw withdrawal latency, paw withdrawal threshold

## Abstract

**Background:**

Neuropathic pain can develop after nerve injury, when deleterious changes occur in injured neurons and glia cells. Melanocortin 4 receptor (MC4R) is involved in the regulation of pain due to its high expressions in brain. Moreover, MC4R could mediate the c-Jun N-terminal kinase (JNK) signaling pathway, but whether the MC4R-regulated JNK signaling pathway participated in neuropathic pain after chronic constriction injury (CCI) is still unclear.

**Methods:**

A total of 128 Sprague-Dawley rats were allocated into four experiment groups: the SHAM group, CCI + NaCl group, CCI + HS group, and CCI + SP + HS group. For the CCI + NaCl group, the sciatic nerves were ligated. For the SHAM group, an identical manner to the CCI without ligation was performed. For CCI + HS and CCI + SP + HS groups, rats were injected with MC4R inhibitor (HS014) and HS014 plus JNK inhibitor (SP600125), respectively, from days 3 to 14 after CCI. Paw withdrawal latency (PWL) and paw withdrawal threshold (PWT) were used to assess the nociceptive behavior. ELISA was used to detect the levels of inflammatory cytokines. qRT-PCR and Western blots (WB) were utilized to examine the mRNA and protein expressions of JNK signaling pathway-related genes. Meanwhile, the expression levels of MC4R and p-JNK were further evaluated by immunohistochemistry (IHC) and immunofluorescence (IF) experiments. Finally, in order to confirm the *in vivo* results, astrocytes were isolated and transfected with MC4R-overexpression plasmid. Furthermore, the protein expressions of JNK signaling pathway-related genes were tested by WB.

**Results:**

It was showed that the values of PWL and PWT were significantly increased in CCI + HS group and CCI + SP + HS group compared with CCI + NaCl group. The increased interleukin-6 (IL-6), IL-1β, and tumor necrosis factor-α (TNF-α) secretion in CCI + NaCl group was lowered by HS and SP + HS. MC4R, p-JNK, ATF3, and c-Jun levels were up-regulated with CCI surgery, but down-regulated with HS and SP + HS treatments. Moreover, the IHC and IF results further revealed that MC4R and p-JNK expressions in CCI + NaCl group were remarkably higher than those in HS group and HS + SP group. *In vitro* data also indicated that HS, SP, and SP + HS could down-regulate the expressions of MC4R, p-JNK, ATF3, and c-Jun in M1830 astrocytes.

**Conclusion:**

Our findings indicated that MC4R is involved in neuropathic pain by regulating JNK signaling pathway after CCI.

## Introduction

Neuropathic pain is defined as pain arising as a direct consequence of a lesion or dysfunction of the somatosensory nervous system and is mediated by a complex interaction of mechanisms in the central nervous systems (CNS) and peripheral nervous systems (PNS) that are among the most intractable of pain syndromes (Lord and Duncan, 1987; Baron, 2009; Cohen and Mao, 2014). It affects a considerable proportion of the human population worldwide and severely affects their emotional well-being and overall quality of life (Baron, 2006). Neuropathic pain is mainly caused by a variety of etiologies, such as toxicity, trauma, infection, ischemia, and heredity, and characterized by increased allodynia and hyperalgesia in response to external stimuli (Birklein, 2002; Ro and Chang, 2005). To date, many drugs have been applied in the clinic, including tricyclic anti-depressants, opioids, and antiepileptics, but there is no effective treatment for releasing for many patients from neuropathic pain and these compounds are accompanied by unacceptable side effects (Rowbotham, 2005; Cohen and Mao, 2014). Hence, the molecular mechanisms underlying the development of neuropathic pain remain need to further explore and a great clinical challenge is to find alternative therapies for the treatment of neuropathic pain. There is increasing evidence which has indicated that spinal neuroinflammation may play a critical role in the pathological states of neuropathic pain. After the nervous system is damaged, the inflammatory cells in CNS or PNS, including microglia and astrocytes, inevitably activates and the inflammatory medium is released which causes an inflammation reaction. Excessive inflammatory reaction is hurtful and stimulating to the neurons (Hossain et al., 2017; Zhang et al., 2017).

Melanocortin receptors (MCRs), including MC1R to MC5R, belonging to a superfamily of seven transmembrane G-protein coupled receptors, are involved in a wide range of physiological functions, including memory and/or learning, thermoregulation, analgesia, stress responses, feeding behavior, inflammation, and pigmentation, etc. (Song et al., 2015; Li et al., 2017). Among the five MCRs, MC4R is expressed primarily in the CNS, and is therefore referred as the neural MCR (Tao, 2010). Besides neurons, astrocytes were also reported to express the MC4R (Selkirk et al., 2007). In recent decades, it has been discovered that an intraplantar injection of MC4R antagonists could weaken chronic constriction injury (CCI)-induced allodynia and hyperalgesia, thereby this research suggested that MC4R might exert an important role in the formation and transduction of neuropathic pain (Starowicz et al., 2004; Chu et al., 2012b). However, the detailed mechanism through which MC4R regulates the process of neuropathic pain is still elusive.

In recent decades, MAPK signaling pathway has been found to regulate neuropathic pain. For example, p38 MAPK activation was one aspect of the signaling cascade that culminated in tumor necrosis factor-α (TNF-α) synthesis and contributed to mechanical allodynia after CCI (Xu et al., 2007). Intrathecal lidocaine could reverse tactile allodynia following CCI through attenuation of p-p38 MAPK in the activated microglia (Gu et al., 2008). Additionally, by using p38 MAPK inhibitor, researchers found that MC4R induced hyperalgesia and allodynia after CCI by the activation of p38 MAPK in dorsal root ganglion (DRG) (Chu et al., 2012b, c). c-Jun N-terminal kinase (JNK) is another branched signaling pathway of MAPK that can be triggered by various stress stimuli and leads to the upregulation of proinflammatory mediators (Liu et al., 2017; Yu et al., 2018). Recently, augmenting reports have suggested that JNK is involved in the regulation of neuropathic pain (Cao et al., 2015; Chen et al., 2016). Moreover, previous studies also verified that MC4R-mediated inflammation reaction could significantly decrease the expression levels of JNK signaling pathway (Chu et al., 2012a; Chen et al., 2018). However, whether MC4R activation can attenuate neuroinflammation and ameliorate neuropathic pain via regulating the JNK signaling pathway is still unclear. Thus, in this study, by using CCI rat models and astrocytes, we explored the role of MC4R in neuropathic pain via affecting the JNK signal transduction pathway.

## Materials and Methods

### Animal Experiments

Specific pathogen-free (SPF) adult male Sprague-Dawley rats (200–250 g), provided by the Experimental Animal Center of Qingdao University, were housed in a standard cage and fed a standard laboratory diet and tap water *ad libitum* in a 12 h light/dark cycle (light on 08:30–20:30) at a constant room temperature (23 ± 1°C). The cages were placed in the experimental room 24 h before the behavioral test to allow for acclimatization. The animals and all surgical and experimental procedures in this study were carried out after approval by the Animal Care and Research Ethics Committee of Qingdao University, in compliance with international laws and policies (Directive, 2010/63/EU of the European parliament and of the council of 22 September 2010 on the protection of animals used for scientific purposes; Guide for the Care and Use of Laboratory Animals, US National Research Council, 2011).

**FIGURE 1:**
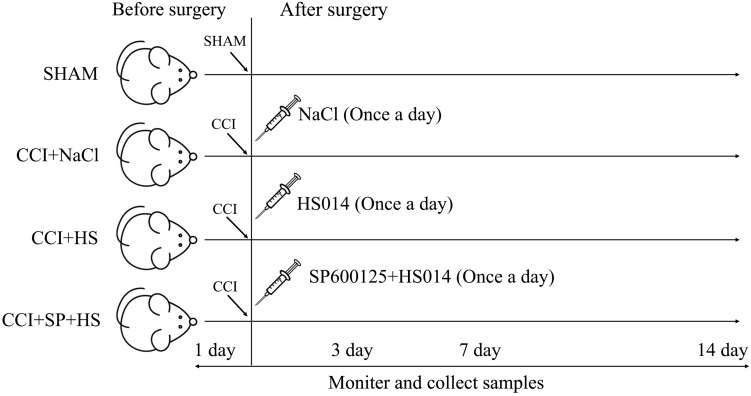
Pattern diagram of animal experiment grouping.

A total of 128 Sprague-Dawley rats were randomly assigned to four groups (*n* = 32 per group): SHAM group, CCI + NaCl group, CCI + HS group, and CCI + SP + HS group. Then, the groups were further divided into four subgroups according to the different time points (1 day before surgery, 3 days after surgery, 7 days after surgery, and 14 days after surgery), with eight rats per subgroup. For the CCI surgery, rats were anesthetized by an intraperitoneal injection of chloral hydrate (40 mg/kg). One side of the common sciatic nerve was exposed at the level of the middle of the thigh by blunt dissection through the biceps femoris. Proximal to the sciatic trifurcation, about 6 mm of nerve was freed of adhering tissue and four ligatures (4.0 chromic gut) were tied loosely around it, with about 1 mm spacing. The length of nerve thus affected was 4–5 mm long. Rats in the SHAM group were operated on in an identical manner to that described for CCI, during which the sciatic nerves were not ligated. Additionally, as shown in Figure 1, rats in the CCI + HS group and CCI + SP + HS group were injected with 1 nmol HS014 (an inhibitor of MC4R, at 50 μl/kg once a day) and HS014 + SP600125 (an inhibitor of JNK, at 10 μg/kg once a day), respectively, from the 3rd day to the 14th day after CCI surgery through an implanted catheter. All efforts were taken to minimize the number of animals used and their suffering. After surgery, rats were housed in cages individually.

### Intrathecal Catheter Insertion

Rats were anesthetized by intraperitoneal injection of 10% chloral hydrate (350 mg/kg). Rats were placed in the prone position following anesthetization. After exposure of the L6 interspinous space and the ligamentum flavum, a polyethylene catheter (PE0503) was inserted into the subarachnoid space at the L5–L6 intervertebral space. The catheter was successfully inserted into the subarachnoid space when the animal exhibited a side tail swing or a hind leg twitch. Thereafter, polyethylene catheter (PE10) was placed in the lumbar spinal cord enlargement (depth 7.0–7.5 cm).

PE10 catheter exposed the skin 3–4 cm. A total of 2% lidocaine (20 μl) was injected through the catheter 3 days following catheter insertion. The present study only included lidocaine-positive rats that exhibited obvious hind limb paralysis in both hind limbs within 30 s following the lidocaine injection. Rats were excluded when obvious limb paralysis or movement disorders following catheter insertion were present, or unilateral limb paralysis occurred following lidocaine injection. The catheter was then flushed with normal saline (10 μl) after the medication injection. In the CCI group, two rats were excluded after intrathecal catheter insertion due to limb paralysis, but another two rats with CCI surgery were immediately supplemented for the intrathecal catheter insertion.

### Drug Administration

Rats in the SHAM and CCI + NaCl groups were intrathecally administered with normal saline (20 μl) once a day from the 3rd day to the 14th day after surgery. Rats in the CCI + HS and CCI + SP + HS groups were administered with HS014 (50 μl/kg) and HS014 + SP600125 (10 μg/kg), respectively, once a day from the 3rd day to the 14th day after surgery.

### Nociceptive Behavior

The behavior testing was performed 30 min after drugs administration and was done within 1 h after administration. Animals were habituated to the testing environment daily for at least 2 days before baseline testing. The nociceptive behavior was monitored 1 day before surgery and 3, 7, and 14 days after surgery and evaluated through the paw withdrawal latency (PWL) in response to radiant heat and paw withdrawal threshold (PWT) in response to mechanical allodynia. The PWT was measured by a PL-20 Plantar Analgesia Tester (Chengdu Technology & Market Co., Ltd.). For testing heat sensitivity, animals were placed in plastic boxes placed on a 6 mm-thick glass plate at room temperature and allowed 10 min for habituation before examination. Then the plantar surface of animals was exposed to a beam of radiant heat (beam diameter 0.5 cm and intensity 20 I.R.) through a transparent glass surface and heat sensitivity expressed as PWL. The baseline latencies were adjusted to 10–14 s with a maximum of 20 s as cut-off to avoid potential tissue damage. The latencies were averaged over three trials, separated by 5 min intervals. For testing mechanical sensitivity, animals were put into the 23905 plantar tests (IITC, United States) with plastic boxes on a metal grid floor (3 mm × 3 mm) and allowed 15 min for habituation before the threshold testing. Mechanical allodynia was tested using von Frey hairs. The plantar surface of the operated paw was stimulated with a series of von Frey hairs with increasing force, presented perpendicular to the plantar surface for 2–3 s, 5 min interval between the tests. Tests were performed for five times and the average was calculated.

### RNA Extraction and qRT-PCR

The samples of lumbar spinal cords (surgical side) were collected 1 day before surgery and 3, 7, and 14 days after surgery to analyze the expressions of *MC4R* and *β-actin* by qRT-PCR. Briefly, rats were anesthetized with sodium pentobarbital (20 mg/kg, i.p.). Then, the skin was cut along the spinous processes, and the lumbar spine was cut off. The lamina and pedicles were removed to expose the spinal cord, and carefully collected the lumbar enlargement (L4–6). Thereafter, the anterior horn of the spinal cord was removed and the DRG (surgical side) was isolated along the L4–6. Total RNA was extracted from the DRG (surgical side) with the use of Trizol, according to the instructions of the supplier (Invitrogen, United States). RNA quality and quantity were measured with a Nanodrop Spectrophotometer, while RNA integrity was assessed by gel electrophoresis. The first-strand complementary DNA (cDNA) was synthesized with 5 μg of the total RNA and 500 ng of random primers using PrimeScript RT Reagent Kit (Takara Bio, Japan). qRT-PCR was performed with SYBR Green PCR mix in an ABI PRISM^®^ 7500 Sequence Detection System (Applied Biosystems, United States). The detector was programmed with the following PCR conditions: pre-denaturation at 95°C for 2 min, 40 cycles of 5 s of denaturation at 95°C and 34 s of amplification at 60°C, followed by a melting curve. β-Actin mRNA expression was used as an internal standard, and the primers used for this were 5′- TCCATCATCATGAAGTGACGT-3′ and 5′-GAGCAATGATCTTGATCTTCAT-3′. The primers used for *MC4R* were 5′-CTTGCACAGTATCGGGCGTTCT-3′ and 5′-GTTCTTGACTCCGCAGGGCATA-3′. Finally, the relative quantification values of these genes were computed with the 2^–△^^△^^Ct^ method by normalizing to β-actin.

### WB Analysis

The DRG (surgical side) were removed 1 day before surgery and 3, 7, and 14 days after surgery after operation once given an overdose of chloral hydrate. To prepare lysates, frozen tissue samples were homogenized in a radioimmunoprecipitation (RIPA) buffer (Beyotime, China) containing a mixture of proteinase and phosphatase inhibitors in ice. The homogenate was centrifuged at 12,000 rpm for 20 min in a microcentrifuge at 4°C, the low speed pellet was discarded and the supernatant was collected. Protein concentration was quantified using a BCA kit (Thermo Fisher Scientific, United States) in accordance with the manufacturer’s instructions. Equivalent amounts of protein (50 μg) from different samples were separated on 10% sodium dodecyl sulfate-polyacrylamide gels for electrophoresis (SDS-PAGE) and transferred onto a polyvinylidene difluoride (PVDF) filter membrane (Thermo Fisher Scientific, United States) by a transfer apparatus at 300 mA for 2 h. The blots were blocked with 5% non-fat dry milk for 120 min and incubated overnight at 4°C with rabbit anti-MC4R (1:1500 dilution; Abcam, United States), anti-p-JNK (1:500 dilution; Abcam, United States), anti-JNK (1:1000 dilution; Abcam, United States), anti-ATF3 (1:1000 dilution; Abcam, United States), anti-c-Jun (1:2000 dilution; Abcam, United States), and anti-GAPDH (1: 5000 dilution; Santa Cruz Biotechnology Inc., United States) antibodies. After being washed with phosphate buffer containing 0.1% Tween (PBST), blots were further incubated with stabilized horseradish peroxidase (HRP)-conjugated goat anti-rabbit IgG (H + L) and goat anti-rat IgG (H + L) secondary antibodies (1:5000 dilution; Zhongshan Gold Bridge Biological Technology Co., China) and left for 1 h at room temperature. Blots were then extensively washed and HRP activity was detected using enhanced chemiluminescence detection system (Beyotime, China). The signal intensity (pixels/mm^2^) quantified (ImageJ, NIH) was normalized to that of GAPDH and eventually quantified with Image-Pro Plus 6.0 software.

### Inflammatory Cytokines Analysis

The DRG (surgical side) was diluted 1:2 (v/v) in sterile saline solution and homogenized with a glass homogenizer. Then the homogenates were centrifuged at 3000 × *g* for 15 min at 4°C and supernatants were collected for the measurement of the concentrations of interleukin-6 (IL-6), IL-1β, and TNF-α by enzyme-linked immunosorbent assay kit (ELISA kit; Nanjing SenBeiJia Biological Technology Co., Ltd., China) as per the manufacturer’s instructions.

### Immunohistochemistry Test

Immunohistochemistry (IHC) was carried out 7 days after surgery. The rats were anesthetized with excessive 10% chloral hydrate solution and perfused transcardially with 250 ml of heparinized PBS, followed by perfusion with 300 ml of 10% formaldehyde solution for 30 min. Then, the rat was decapitated and the DRG (surgical side) was removed immediately, immersed continuously in the 4% formaldehyde solution at 4°C overnight until tissue sinking to the bottom, and embedded with paraffin wax. The paraffin-embedded tissue arrays were performed in 4-μm sections and deparaffinized in xylene and rehydrated with a graded alcohol solution. The sections were soaked in 0.01 M citrate buffer (pH 6.0) and heated in a microwave oven for hot repair antigen. After washing twice in PBS for 2 min each, endogenous peroxidase activity was blocked using 0.3% hydrogen peroxide (H_2_O_2_). Next, the sections were incubated with primary antibodies: MC4R (1:150 dilution; Abcam, United States) and p-JNK (1:200 dilution; Abcam, United States), at 4°C overnight and washed twice with PBS for 2 min each. Eventually, the sections were exposed to biotinylation conjugated secondary antibody (1:1000 dilution; Invitrogen, United States) at 37°C for 1 h. Followed by PBS washing, immunostaining was visualized with diaminobenzidine (DAB). The images (200×) were captured using microscope (Shanghai Optical Instrument Factory No. 1, China).

### Immunofluorescence Staining

Transverse sections (4 μm) of the DRG (surgical side) from different groups at postoperative day 7 were fixed in 4% paraformaldehyde, permeabilized with 0.1% Triton X-100, and blocked in 10% normal serum (species the same as the secondary antibody) for 2 h at room temperature to avoid unspecific staining. Next, the sections were incubated with primary antibody against p-JNK (1:200 dilution; Abcam, United States) at 4°C overnight. After being washed with PBS twice for 5 min each, the sections were incubated with fluorescein isothiocyanate (FITC) conjugated secondary antibody (1:1000 dilution; Invitrogen, United States) at 37°C for 1 h in the dark. Ultimately, followed by rinsing with PBS twice for 5 min each, the sections were counterstained with DAPI (Beyotime, Nantong, China) and examined under a confocal microscope (Leica, Germany) with appropriate excitation and emission filters to acquire representative images.

### Astrocyte Culture and Treatments

Mouse spinal cord astrocytes cell line M1830 was purchased from SXBIO Co., Ltd. (Shanghai, China). Matrigel was diluted by fresh DMEM/F12 medium at 1:30. Thereafter, 2 ml diluted matrigel was added into the 10 cm-culture dish to cover the bottom. Culture dish was coated with matrigel for 30 min under humidified 5% CO_2_ and 95% O_2_ at 37°C. Then, discarded the diluted matrigel and sealed the culture dish with parafilm. Frozen M1830 cells were unfreezed in 37°C water bath. Then, added the M1830 cells in the coated culture dish with 9 ml astrocyte medium. M1830 cells were cultured under humidified 5% CO_2_ and 95% O_2_ at 37°C, refed every 2 days, and passaged every 4–6 days.

The MC4R sequence was synthesized according to the full-length MC4R sequence and then subcloned into a pCDNA3.1 vector (Invitrogen, China). The empty pcDNA3.1 vector was used as the negative control (NC). M1830 were further transfected with MC4R-overexpression plasmid or NC plasmid using Lipofectamine 2000 (Promega, United States) according to the manufacturer’s protocols. Afterward, astrocytes, MC4R-overexpressed astrocytes, and NC astrocytes were treated with HS014 and SP600125 as described in animal experiments to further explore the role of MC4R in neuropathic pain by JNK signaling.

### Statistical Analysis

In order to ensure the reliability and authenticity of the experimental data, all of the tissues and biochemical indexes were obtained from ipsilateral ones (surgical side). All data are represented as mean ± standard deviation (SD) from at least triplicate experiments. Results were analyzed by SPSS 19.0 software (SPSS Inc., United States). A one-way analysis of variance (ANOVA) was used to determine significant differences between groups, and the individual comparisons were performed with Tukey’s *post hoc* test. A two-sided *P*-value of <0.05 was considered to indicate a statistically significant difference.

## Results

### MC4R Antagonist and JNK Inhibitor Alleviates Radiant Heat and Mechanical Allodynia Induced by CCI Surgery

**FIGURE 2:**
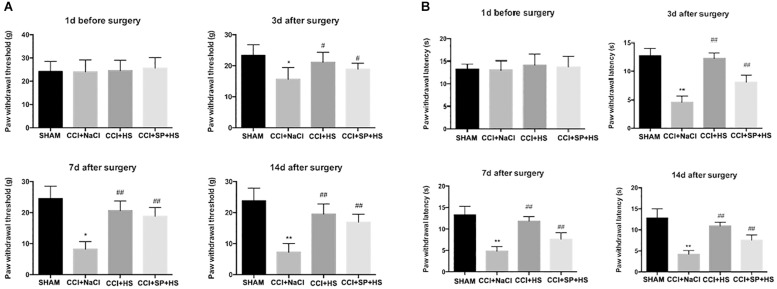
Effects of MC4R antagonist and JNK inhibitor on behavioral mechanical allodynia **(A)** and thermal hyperalgesia **(B)** at day 1 before CCI surgery and at days 3, 7, and 14 after CCI surgery. Results are given as mean ± *SD* (*n* = 8 for each time point in each group). ^∗^*P* < 0.05 and ^∗∗^*P* < 0.01 compared to the SHAM group, ^#^*P* < 0.05 and ^##^*P* < 0.01 compared to the CCI + NaCl group.

As shown in Figure 2, the values of PWL and PWT were significantly decreased after CCI surgery compared to SHAM group (*P* < 0.01). However, when giving MC4R antagonist and JNK inhibitor, the values of PWL and PWT at days 3, 7, and 14 after CCI surgery were significantly increased compared with that in CCI + NaCl group (*P* < 0.05). Therefore, these results suggested that MC4R antagonist and JNK inhibitor might relieve pain-related behavioral changes of CCI rats. Moreover, MC4R antagonist and JNK inhibitor were considered to involve in the alterations of nociceptive behavior in CCI rats, so it was speculated that MC4R and JNK might play important roles in CCI neuropathic pain rats.

### MC4R Participates in the CCI-Induced Inflammation

**FIGURE 3:**
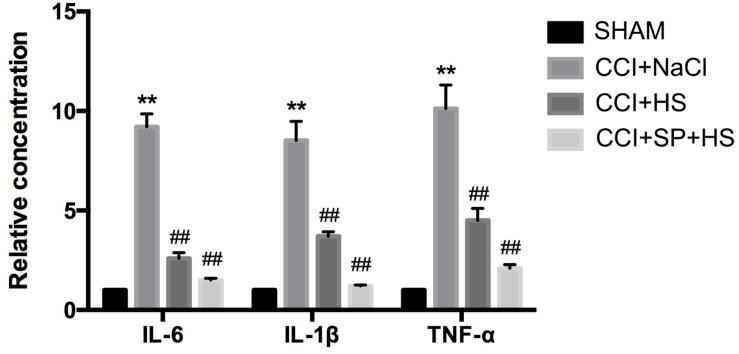
The expressions of inflammatory cytokines in the lumbar spinal cords of rats by ELISA on day 14 after CCI surgery. Results are given as mean ± *SD* (*n* = 8 for each group). ^∗∗^*P* < 0.01 compared to the SHAM group, ^##^*P* < 0.01 compared to the CCI + NaCl group.

At day 14 after surgery, the inflammation level of lumbar spinal cords (surgical side) was detected by ELISA. Compared with the SHAM group, rats with CCI surgery showed higher levels of IL-6, IL-1β, and TNF-α (*P* < 0.01). However, CCI + HS and CCI + SP + HS treatments significantly decreased the secretions of IL-6, IL-1β, and TNF-α in comparison to that in CCI + NaCl group (*P* < 0.01) (Figure 3). The above data indicated that MC4R and JNK might contribute to the inflammation of CCI neuropathic pain rats.

### The Expressions of MC4R and JNK Signaling After CCI Surgery

**FIGURE 4:**
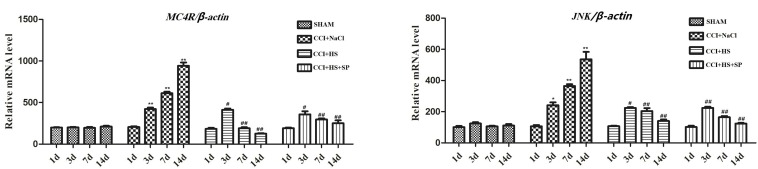
The mRNA expression of *MC4R* and *JNK* in the lumbar spinal cords of rats by qRT-PCR on 1 day before CCI surgery and 3, 7, and 14 days after CCI surgery. Results are given as mean ± *SD* (*n* = 8 for each time point in each group). ^∗^*P* < 0.05 and ^∗∗^*P* < 0.01 compared to the SHAM group, ^#^*P* < 0.05 and ^##^*P* < 0.01 compared to the CCI + NaCl group.

**FIGURE 5:**
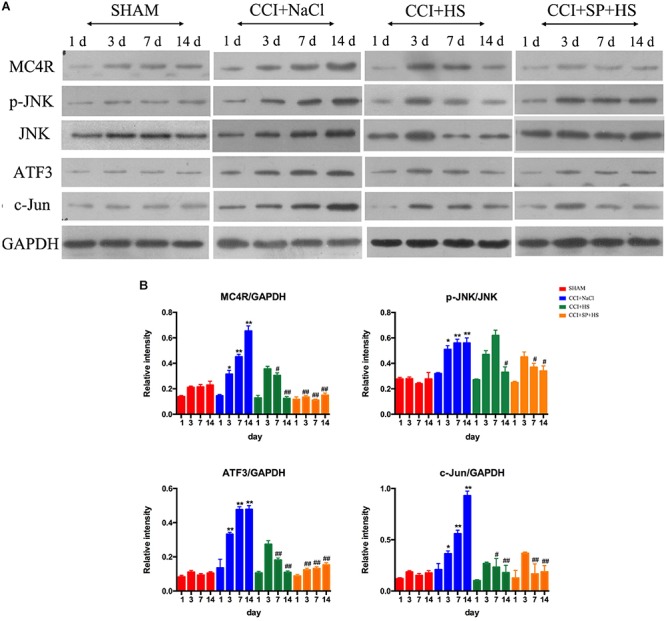
Effects of MC4R antagonist and JNK inhibitor on the expressions of MC4R and key proteins in JNK signaling pathway. **(A)** Expressions of MC4R, p-JNK, JNK, ATF3, c-Jun, and GAPDH in the lumbar spinal cords of rats on 1 day before CCI surgery and 3, 7, and 14 days after CCI surgery. **(B)** The ratio of MC4R, p-JNK, ATF3, or c-Jun to GAPDH was analyzed by Image J. Results are given as mean ± *SD* (*n* = 8 for each time point in each group). ^∗^*P* < 0.05 and ^∗∗^*P* < 0.01 compared to the SHAM group, ^#^*P* < 0.05 and ^##^*P* < 0.01 compared to the CCI + NaCl group.

**FIGURE 6:**
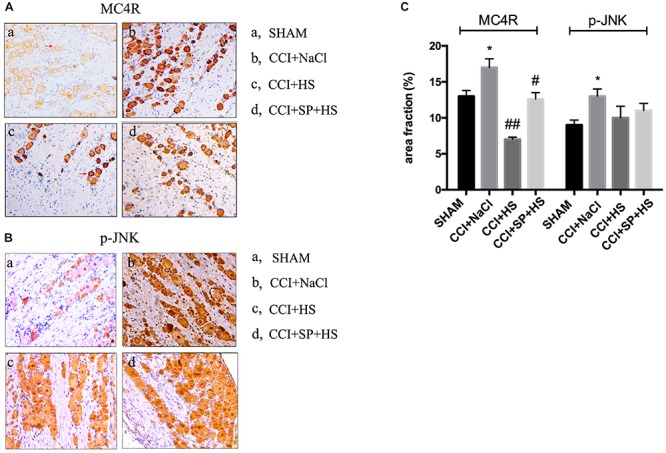
Localization study of MC4R and p-JNK in the lumbar spinal cords of CCI rats with MC4R antagonist and JNK inhibitor after 7 days was evaluated by IHC (200×). **(A)** MC4R level. **(B)** p-JNK level. **(C)** Quantification of IHC staining-positive area by Image J. The red arrows indicate IHC staining positive cells. Results are given as mean ± *SD* (*n* = 8 for each group). ^∗^*P* < 0.05 compared to the SHAM group, ^#^*P* < 0.05 and ^##^*P* < 0.01 compared to the CCI + NaCl group.

Melanocortin 4 receptor and JNK signaling-related genes were assessed at 1 day before surgery and 3, 7, and 14 days after CCI surgery by qRT-PCR and WB. The up-regulated *MC4R* and *JNK* mRNA (*P* < 0.01) (Figure 4), and increased MC4R, p-JNK, ATF3, and c-Jun protein levels (*P* < 0.05) were observed on days 3, 7, and 14 days after CCI surgery (Figure 5). Furthermore, these expressions were gradually augmented over time in the CCI + NaCl group, with the peak on postoperative day 14 when compared with the SHAM group (Figures 3, 4). However, after administrating HS and SP + HS, the mRNA level of *MC4R* was significantly decreased compared with that in CCI + NaCl group at day 3 (*P* < 0.05), 7 (*P* < 0.01), and 14 (*P* < 0.01), respectively (Figure 4). Moreover, the protein expressions of MC4R, ATF3, and c-Jun were also significantly down-regulated in the CCI + HS group compared to that in the CCI + NaCl group at days 7 and 14 after surgery (*P* < 0.05), but HS also obviously decreased c-Jun and p-JNK levels at days 3 and day 14, respectively, after surgery (*P* < 0.05). In addition, with SP + HS administration, MC4R and ATF3 expressions were significantly decreased at day 3, 7, and 14 after surgery compared with that in CCI + NaCl group (*P* < 0.01). Furthermore, SP + HS also down-regulated p-JNK (*P* < 0.05) and c-Jun (*P* < 0.01) expressions at days 7 and 14 after surgery compared to CCI + NaCl group (Figure 5). In addition, IHC and immunofluorescence (IF) were further conducted to evaluate the expressions of MC4R and p-JNK. As illustrated in Figure 6, it was clearly seen that MC4R and p-JNK expressions were remarkably elevated in the CCI + NaCl group in comparison with the SHAM group (*P* < 0.05), but the expressions of MC4R in the CCI + HS and CCI + SP + HS groups were markedly lower than those in CCI + NaCl group (*P* < 0.05). Besides, although HS and SP + HS lowered p-JNK levels to some extent, no significant differences were noticed. Moreover, the results also exhibited the same trend as IHC (Supplementary Figure S1). Hence, these data indicated that MC4R and JNK signaling might participant in the formation of neuropathic pain in CCI rats and the neuropathic pain in CCI rats might be relieved via MC4R regulating the JNK signaling.

### MC4R Is Related to Neuron Injury by Regulating JNK Signaling *in vitro*

**FIGURE 7:**
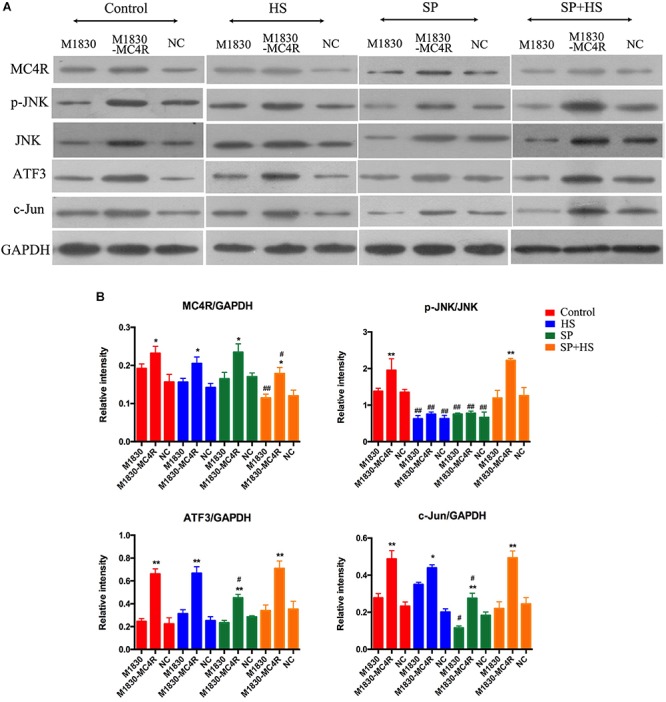
The influences of MC4R antagonist and JNK inhibitor on the protein expressions of MC4R, p-JNK, ATF3, and c-Jun in MC4R-overexpressed astrocytes. **(A)** Expressions of MC4R, p-JNK, JNK, ATF3, c-Jun, and GAPDH. **(B)** The ratio of MC4R, p-JNK, ATF3, or c-Jun to GAPDH was analyzed by Image J. Results are given as mean ± *SD* (*n* = 8 for each group). ^∗^*P* < 0.05 and ^∗∗^*P* < 0.01 compared to the M1830 cells within groups, ^#^*P* < 0.05 and ^##^*P* < 0.01 compared to the control group.

In order to further explore the role of MC4R in the generation of neuropathic pain, MC4R-overexpression plasmid was transfected into astrocytes. It was discovered that MC4R overexpression could effectively elevate the expressions of MC4R (*P* < 0.05), p-JNK (*P* < 0.01), ATF3 (*P* < 0.01), and c-Jun (*P* < 0.01) (Figure 7). Moreover, HS and SP obviously decreased the phosphorylation level of JNK in wild-type M1830 and M1830 transfected with MC4R plasmid as well as NC plasmid (*P* < 0.01). What’s more, SP + HS significantly decreased MC4R expressions in wild-type (*P* < 0.01) and MC4R-overexpression M1830 cells (*P* < 0.05). In addition, SP significantly decreased the levels of ATF3 in MC4R-overexpression M1830 cells (*P* < 0.05) and down-regulated c-Jun expressions in both wild-type and MC4R-overexpression M1830 cells (*P* < 0.05). Thus, these findings further verified that MC4R might take part in neuron injury by regulating JNK signaling.

## Discussion

The CCI model is a rat model of neuropathic pain based on a unilateral loose ligation of the sciatic nerve (Baad-Hansen and Benoliel, 2017). Chronic neuropathic pain resulting from peripheral nerve injury is characterized by pathological symptoms, such as hyperalgesia and allodynia to mechanical and thermal stimuli, as well as spontaneous pain (Koltzenburg, 1998; Vry et al., 2004). Thus, in the present study, we first assessed the effects of HS and SP + HS on the nociceptive behavior of CCI surgery rats using PWT and PWL. Here, the decreased PWT and PWL values in CCI + NaCl group were augmented by HS and SP + HS, indicating that both HS and SP possessed a potent antinociceptive effect after CCI. Moreover, increasing evidence indicates that spinal neuroinflammation may play a critical role in the pathological states of neuropathic pain (Sindrup and Jensen, 1999; Martini et al., 2016). Therefore, the levels of pro-inflammatory cytokines, such as IL-6, IL-β, and TNF-α, were measured by ELISA. As expected, these cytokines were induced by CCI surgery, but decreased by HS and SP + HS at day 14 after CCI surgery. JNK signaling pathway contributes to inflammation and can be activated by pro-inflammatory cytokines (Yadav and Weng, 2017). Thus, there is no doubt that the administration of JNK inhibitor was able to decrease the inflammation in lumbar spinal cords. Additionally, JNK is also essential for chronic inflammatory pain and can be persistently activated in spinal cord after nerve injury (Chen et al., 2015). Therefore, the expressions of MC4R and related proteins in the JNK signaling pathway were examined in order to figure out the underlying molecular mechanisms. Similar to previous researches (Chu et al., 2012b; Jiang et al., 2019), data in the current study also showed that MC4R level was significantly induced with CCI surgery, but decreased by HS and SP treatments after CCI surgery, both on mRNA level and protein level. More recently, there has been interest in the c-Jun-related bZIP transcription factor, ATF3, and its function in neurons. ATF3 can be used as a marker of axonal injury (Chu et al., 2012a). During CCI surgery, ATF3 expression was also found to be increased in many studies (Kernisant et al., 2008; Allchorne et al., 2012). Similar to the expression of MC4R, in the present study, the expressions of p-JNK, ATF3, and c-Jun were also up-regulated with CCI surgery, but down-regulated after administration of HS and SP. Moreover, by performing IHC and IF experiments, the expressions of MC4R and JNK signaling pathway-related proteins in lumbar spinal cords were further verified. Taken together, the above findings suggested that the JNK signaling pathway activation was prevented by blocking MC4R with its antagonist and played a key role in antinociception.

Based on the *in vivo* results, the *in vitro* experiment (tissues from the surgical sides) was often carried out to confirm the effects of MC4R and JNK signaling pathway in neuropathic pain (Obata et al., 2003; Ikeda et al., 2012; Jha et al., 2012). A large number of studies have elucidated that glia-mediated inflammatory reactions play a pivotal role in the generation and development of persistent pain, such as inflammatory pain, neuropathic pain, and bone cancer pain. Gila cells mainly include microglia and astrocytes in the spinal cord, which are the guarders of the brain and could exert immune defense role in CNS (Vallejo et al., 2010; Qian et al., 2016). Moreover, microglia is responsible for the initiation of neuropathic pain, whereas astrocytes are in charge of the maintenance of neuropathic pain. Therefore, MC4R-overexpressed astrocytes M1830 were used for further confirmation in the current study. In the *in vitro* experiments, HS, SP, and SP + HS were added into wild-type and MC4R-overexpression M1830 cells, respectively. With MC4R overexpression, the expressions of MC4R, p-JNK, ATF3, and c-Jun were significantly increased. Moreover, similar with the results in rats, HS decreased p-JNK level, and SP + HS decreased MC4R expression, while SP decreased ATF3 and c-Jun levels compared with the control group, demonstrating that MC4R might take part in neuron injury by regulating JNK signaling.

In summary, the present study revealed that CCI-induced neuropathic pain increased MC4R expression and activated JNK signaling pathway. However, microinjection of MC4R antagonist HS014 and JNK inhibitor SP600125 effectively attenuated CCI-induced thermal hyperalgesia and mechanical allodynia. At the same time, the JNK signaling pathway was also inhibited when giving HS or SP after CCI surgery. Thus, our findings implied that the therapeutic mechanism of MC4R antagonist in neuropathic pain is due to its suppression of JNK and related inflammation. In conclusion, this study shows that MC4R can promote descending facilitation of neuropathic pain through regulating the JNK signaling pathway.

## Data Availability

The raw data supporting the conclusions of this manuscript will be made available by the authors, without undue reservation, to any qualified researcher.

## Ethics Statement

All surgical and experimental procedures in this study were carried out after approval by the Animal Care and Research Ethics Committee of Qingdao University, in compliance with international laws and policies (Directive, 2010/63/EU of the European parliament and of the council of 22 September 2010 on the protection of animals used for scientific purposes; Guide for the Care and Use of Laboratory Animals, US National Research Council, 2011).

## Author Contributions

HC designed the study and drafted the manuscript. YZ performed the experiments and collected the data. YX analyzed the data and edited the language of the manuscript.

## Conflict of Interest Statement

The authors declare that the research was conducted in the absence of any commercial or financial relationships that could be construed as a potential conflict of interest.

## Supplementary Material

FIGURE S1 The lumbar spinal cords’ sections were obtained from rats with CCI after 7 days to assess the expression of p-JNK through IF technology. Results are given as mean ± *SD* (*n* = 8 for each group). ^∗∗^*P* < 0.01 compared to the SHAM group, ^#^*P* < 0.05 compared to the CCI + NaCl group.
