# Supplementary figures and images for: MC4R Is Involved in Neuropathic Pain by Regulating JNK Signaling Pathway After Chronic Constriction Injury
Source: Front Neurosci. 2019 Sep 10;13:919. doi: 10.3389/fnins.2019.00919 (PMC6746920; doi:10.3389/fnins.2019.00919)

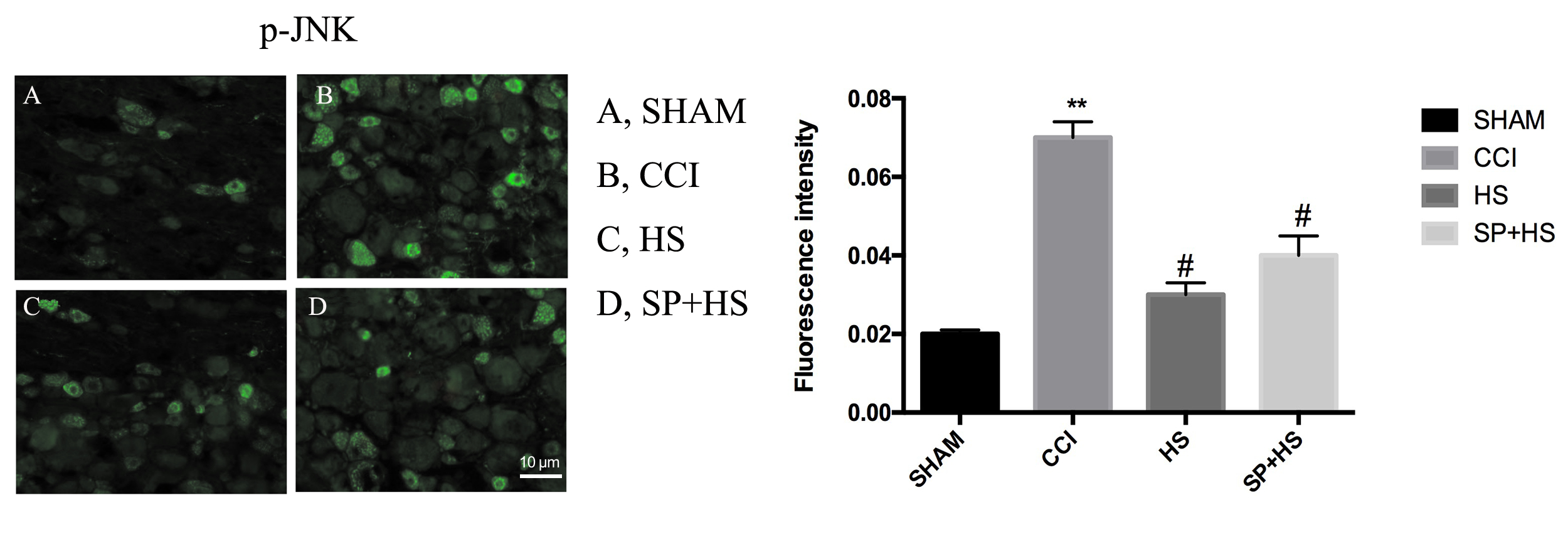

Supplement: FIGURE S1 — The lumbar spinal cords’ sections were obtained from rats with CCI after 7 days to assess the expression of p-JNK through IF technology. Results are given as mean ± SD (n = 8 for each group). ∗∗P < 0.01 compared to the SHAM group, #P < 0.05 compared to the CCI + NaCl group. [file Image_1.JPEG]
